# Explainable Deep Learning for Personalized Age Prediction With Brain Morphology

**DOI:** 10.3389/fnins.2021.674055

**Published:** 2021-05-28

**Authors:** Angela Lombardi, Domenico Diacono, Nicola Amoroso, Alfonso Monaco, João Manuel R. S. Tavares, Roberto Bellotti, Sabina Tangaro

**Affiliations:** ^1^Dipartimento di Fisica, Universitá degli Studi di Bari Aldo Moro, Bari, Italy; ^2^Istituto Nazionale di Fisica Nucleare, Sezione di Bari, Bari, Italy; ^3^Dipartimento di Farmacia - Scienze del Farmaco, Università degli Studi di Bari Aldo Moro, Bari, Italy; ^4^Instituto de Ciência e Inovação em Engenharia Mecânica e Engenharia Industrial, Departamento de Engenharia Mecânica, Faculdade de Engenharia, Universidade Do Porto, Porto, Portugal; ^5^Dipartimento di Scienze del Suolo, della Pianta e degli Alimenti, Università degli Studi di Bari Aldo Moro, Bari, Italy

**Keywords:** explainable artificial intelligence, XAI, brain aging, deep neural networks, machine learning, MRI, FreeSurfer, morphological features

## Abstract

Predicting brain age has become one of the most attractive challenges in computational neuroscience due to the role of the predicted age as an effective biomarker for different brain diseases and conditions. A great variety of machine learning (ML) approaches and deep learning (DL) techniques have been proposed to predict age from brain magnetic resonance imaging scans. If on one hand, DL models could improve performance and reduce model bias compared to other less complex ML methods, on the other hand, they are typically black boxes as do not provide an in-depth understanding of the underlying mechanisms. Explainable Artificial Intelligence (XAI) methods have been recently introduced to provide interpretable decisions of ML and DL algorithms both at local and global level. In this work, we present an explainable DL framework to predict the age of a healthy cohort of subjects from ABIDE I database by using the morphological features extracted from their MRI scans. We embed the two local XAI methods SHAP and LIME to explain the outcomes of the DL models, determine the contribution of each brain morphological descriptor to the final predicted age of each subject and investigate the reliability of the two methods. Our findings indicate that the SHAP method can provide more reliable explanations for the morphological aging mechanisms and be exploited to identify personalized age-related imaging biomarker.

## 1. Introduction

Brain age prediction has become a challenging topic in computational neuroscience, due to the strong link between aging processes and several brain disorders and diseases (Franke and Gaser, 2012; Gaser et al., 2013; Koutsouleris et al., 2014; Cole and Franke, 2017b; Wang et al., 2019). Accordingly, accurate age prediction models measuring the difference between the chronological age and the predicted brain age (i.e., the age gap) have been developed to help identifying novel functional and structural biomarkers for such diseases and provide systems for early diagnosis (Cole et al., 2015, 2019; Cole and Franke, 2017a). In particular, machine learning (ML) and deep learning (DL) algorithms have been successfully applied to predict age from brain MRI scans. Two main approaches are largely adopted to perform brain age prediction: on one hand, a number of selected features such as morphological descriptors, graph-based or other imaging-related features can be extracted from imaging to train different models (Erus et al., 2015; Amoroso et al., 2018, 2019; Bellantuono et al., 2020; Han et al., 2020); on the other hand, more complex models such as convolutional neural networks directly exploiting raw image as input have proven to be particularly effective in brain age prediction even in broad age ranges (Cole et al., 2017, 2019; Feng et al., 2020; Levakov et al., 2020; Peng et al., 2021). Although convolutional neural networks offer undoubted advantages such as reduced preprocessing time and high performance (Cole et al., 2017), both ML and DL feature-based learning approaches based on morphological features are still widely adopted by scientific communities as they allow to investigate the morphological age-related brain changes in a great variety of disorders and conditions (Van Rooij et al., 2018; Corps and Rekik, 2019; Boedhoe et al., 2020; Han et al., 2020).

Several works have shown that DL models improve performance and reduce model bias compared to other less complex ML methods (Couvy-Duchesne et al., 2020; Da Costa et al., 2020; Lombardi et al., 2020c); however, current DL approaches applied to neuroimaging typically do not provide an in-depth understanding of the underlying mechanisms and how they contributed to the outcome. Understanding how the models affect the decisions and how each feature is related to the outcomes can increase confidence in the models and broaden their applications in the clinical setting (Carvalho et al., 2019; Holzinger et al., 2019). In order to overcome these limitations, new explainable methods have been introduced in the last 5 years. Explainable Artificial Intelligence (XAI) is a relatively new field of Artificial Intelligence and it comprises a large amount of techniques that combines ML algorithms with explanatory techniques to develop explainable solutions that have been extensively applied in different domains (Gunning, 2017; Adadi and Berrada, 2018; Biecek, 2018; Guidotti et al., 2018; Miller, 2019; Arrieta et al., 2020; Bussmann et al., 2020). Recent work has suggested that XAI methods constitute a fundamental pillar for personalized medicine, including individualized interventions and targeted treatments (Vu et al., 2018; Fellous et al., 2019; Langlotz et al., 2019). Most widespread explainable techniques comprise local model-agnostic methods that focus on explaining individual predictions of any ML models, such as LIME (Ribeiro et al., 2016, 2018) and SHAP (Lundberg and Lee, 2017). These methods aim at estimating the contribution of individual features toward a specific prediction by perturbing a given instance and observing the effect of these perturbations on the output of the model.

However, as far as we know, there has been little analysis of the reliability and robustness of the explanation methods in computational neuroscience, making their utility for critical applications unclear. In this work, we present an explainable DL framework to predict the age of a healthy cohort of subjects from ABIDE I database (Di Martino et al., 2014) by using morphological features extracted from their MRI scans. We embed two local XAI methods to explain the outcomes of the DL models and determine the contribution of each brain morphological descriptor to the final predicted age of each subject. We propose a complete architecture to compare the two methods, determine their reliability and to extract information on the importance of the most age-related morphological descriptors in order to encourage the use of DL models in clinical settings.

## 2. Materials

### 2.1. Subjects

In this study, we exploited the same dataset used in our previous work (Lombardi et al., 2020b). In particular, we selected *T* = 378 T1-weighted MRI publicly available scans of a cohort of typically-developing individuals from the Autism Brain Imaging Data Exchange (ABIDE I) collected from 17 international sites. The T1-weighted MRI scans were collected with 3 Tesla scanners with different characteristics such as manufacturers and parameters (e.g., echo time, repetition time, flip angle, and field of view). More details about images and acquisition protocols from each site are available at the web page of the initiative^1^. All participating sites received local Institutional Review Board approval for acquisition of the contributed data. Only male subjected were considered in our analysis due to the high imbalance between male and female subjects in the ABIDE data sample. Additionally, we used the full IQ (FIQ) test scores from the phenotype information file and the Signal to Noise Ratio (SNR) from the anatomical quality assessment metrics provided by the publicly available ABIDE Preprocessed repository (Craddock et al., 2013). The SNR was computed as the mean intensity within gray matter divided by the standard deviation of the values outside the brain (Magnotta et al., 2006). The demographic and imaging-related characteristics of the studied subjects are listed in Table 1 for each of the 17 sites.

**Table 1:** Demographic and imaging-related information of the subjects per site.

**Site**	**Samples**	**Age range (years)**	**FIQ (mean ± std)**	**SNR (mean ± std)**
CMU	2	21−25	109.5 ± 0.7	42.5 ± 6.4
KKI	23	8−13	112.9 ± 9.4	23.9 ± 7.8
Leuven 1	13	18−29	116.5 ± 12.7	16 ± 1.7
Leuven 2	14	12−17	NA	13.5 ± 1.7
MaxMun	24	7−48	112.1 ± 8.8	20.2 ± 3.5
NYU	77	6−32	113.7 ± 12.6	12.6 ± 1.6
Olin	13	10−23	116.3 ± 17.0	18.3 ± 2.5
Pitt	22	12−33	110.4 ± 8.3	9.4 ± 1.6
SBL	14	20−42	NA	6.3 ± 1.2
SDSU	14	12−17	110.5 ± 10.3	20.5 ± 4.6
Trinity	25	12−25	110.8 ± 12.2	11.3 ± 2.9
UCLA 1	28	9−18	104.6 ± 10.6	13.8 ± 1.9
UCLA 2	11	10−14	113.1 ± 11.4	13.8 ± 2.1
UM 1	32	8−19	109.8 ± 8.7	22.7 ± 6.5
UM 2	17	13−29	110.3 ± 10.2	24.3 ± 5.0
USM	43	8−40	115.1 ± 13.7	20.5 ± 2.0
Yale	6	8−17	108.1 ± 13.3	21.5 ± 10.8
Total	378	6−48	112.1 ± 11.7	16.6 ± 6.6

### 2.2. Morphological Features

As in our previous work (Lombardi et al., 2020b), the T1 raw scans were preprocessed by using the recon-all pipeline from the software FreeSurfer v.5.3.0 (Dale et al., 1999; Fischl et al., 1999, 2002) on ReCaS datacenter^2^ (Lombardi et al., 2019). The recon-all pipeline allows to segment the brain into 68 cortical regions and 40 sub-cortical region by means of the Desikan–Killiany atlas (Desikan et al., 2006) and Aseg Atlas (Fischl et al., 2002). The output of the pipeline consists in several statistical morphological features related to surface, curvature, thickness and white matter volumes of the cortical regions and volumes of the sub-cortical regions as well as some global brain metrics including surface and volume statistics of each hemisphere, total cerebellar gray and white matter volume, brainstem volume, corpus callosum volume, white matter hypointensities. More details about the steps performed can be found at the web page of the pipeline^3^ and in our previous work (Lombardi et al., 2020b). We constructed the matrix of the features of dimension *T* × *P* with *T* = 387, and *P* = 1, 213, where each row represents a single subject described by *P* morphological features.

## 3. Methods

In this study, we developed a DL framework to:

- Predict the brain age of a healthy cohort of subjects by using their morphological features and DNN models;
- Exploit two local XAI methods to extract personalized age-related features;
- Investigate the reliability of these individual age-related features;
- Compare the two XAI methods.

**Figure 1:**
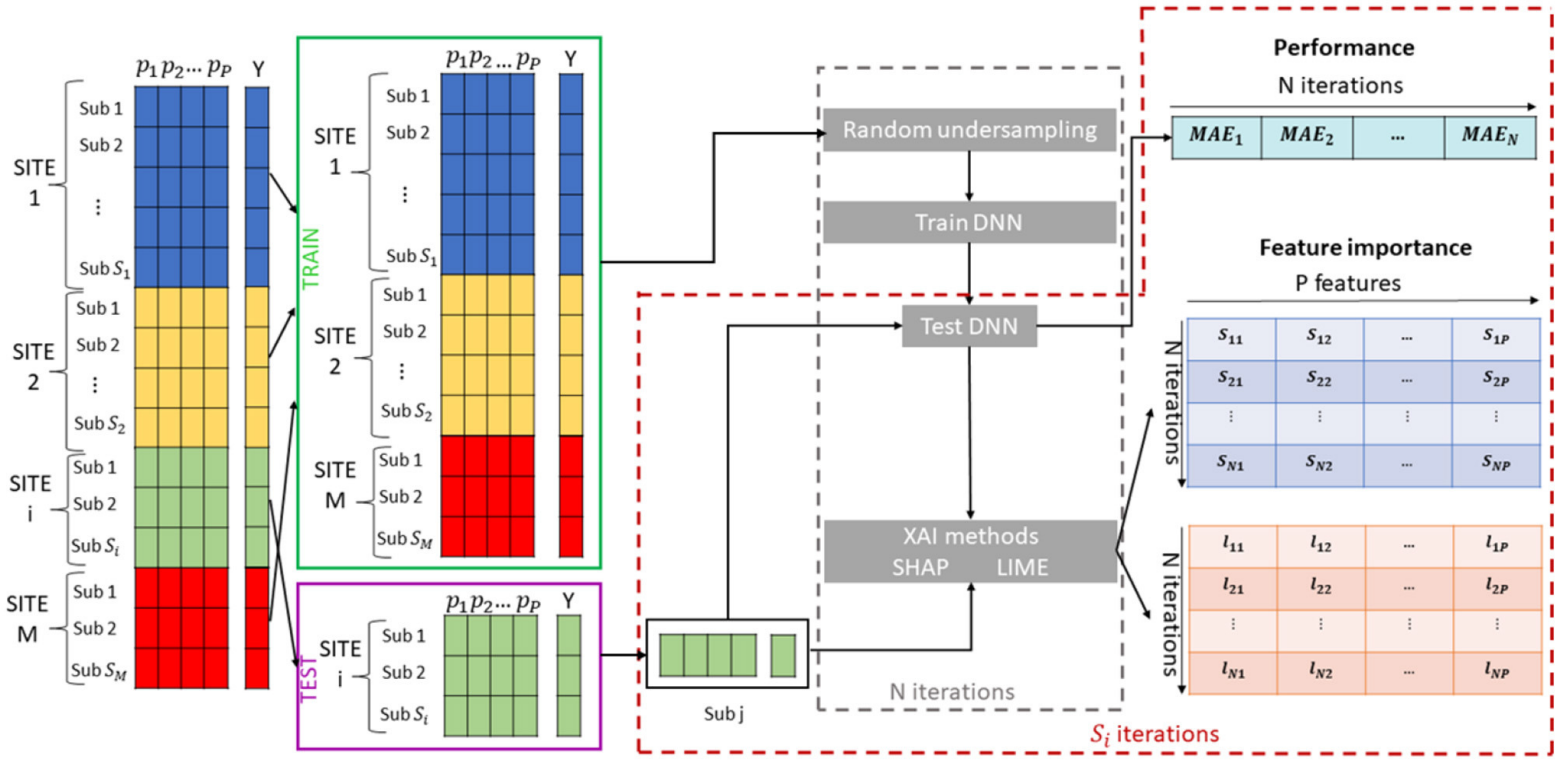
Schematic overview of the Explainable DL framework for brain age prediction.

The overall proposed framework is shown in Figure 1. We adopted a leave-one-site cross validation regression scheme: the data from one site are used as a test set to evaluate the performance of the model while the data from all the other sites are used as training set. This cross-validation scheme has been extensively used in multisite studies as it is possible to test the generalization of the models to a new site, and to investigate the correlation between the variability of the characteristics of the different sites and the performance of the models (Abraham et al., 2017; Bhaumik et al., 2018; Heinsfeld et al., 2018). Since in general the ML algorithms can be sensitive with respect to changes in the training set, returning both the performance and the feature ranking varying from round to round, for each cross-validation round, we randomly under-sampled the training set *N* = 100 times by selecting the 80% of the samples to produce small variations of the composition of the set and for each iteration we trained a DNN model to predict the chronological age of the subjects *Y*, using a fixed percentage of the samples to perform the tuning of the parameters. We tested the DNN models on each sample of the test set collecting *N* = 100 performance MAE values for each subject. Moreover, we applied both SHAP and LIME algorithms to extract the age-related feature importance vector for each subject collecting the two matrix *S* and *L* of dimension [*N* × *P*], whose generic element *s*_*nk*_ (*l*_*nk*_) indicates the SHAP (LIME) value for the *k* feature within the *n* iteration. Accordingly, we analyzed the resulted matrices to investigate the effect of the variability of the training set on both performance and age-related feature importance at subject-level and across subjects. In the following sections, each step of the algorithm is further explained.

### 3.1. Deep Neural Networks

We developed a fully connected DNN architecture. The model and all the computation was implemented using Tensorflow 2.0 (Abadi et al., 2016), with the serial interface. The Input layer shape was composed by 1, 213 units, i.e., the number of features that characterize each subject.

It is well-known that there is no general rule to determine the model hyper-parameters, so we tuned them with a series of 10-fold Grid Search cross validations on training sets, using the left out site as a completely independent test set. In each training the decrease of the loss was monitored using the Keras callback functions EarlyStop, with *patience* = 20, and ModelCheckpoint, in order to stop the training before overfitting. The parameters determined with cross validations were: the activation function (we checked ReLu and tanh), the dropout rate (0, 0.1, 0.2, 0.3), the number of neurons (128, 256, 512, 1, 024), the net optimizer (SGD, Adam), the loss function (Huber and MSE), the learning rate (1*e* − 4, 5*e* − 5, 1*e* − 5), the number of layers (3, 4, 5) and the batch size (20, 100, 400). We reached the final configurations with 4-layers with 512 units per layer, relu as activation function, the SGD optimizer with learning rate 5*e* − 5 and momentum 0.9, the loss function Huber and dropout 0. The number of epochs of each training round was controlled by the trend of the loss function on the validation subset through the callbacks mentioned above. The output layer had a single unit with no activation function, in order to perform the required regression.

The performance of the models were evaluated by means of the Mean Absolute Error (MAE):

(1) $$\begin{array}{l} MAE=\frac{1}{t}\overset{t}{\underset{i=1}{\sum }}|y_{i}-\hat{y_{i}}|, \end{array}$$

with *t* being the sample size for the specific test site, *y*_*i*_ the chronological age, and $\hat{y_{i}}$ the predicted brain age. The correlation coefficient between the chronological age and the predicted age of the subjects was also computed to assess the performance of the models over the whole dataset:

(2) $$\begin{array}{l} R=\frac{\sum_{i=1}^{T}\left(y_{i}-ȳ\right)\left(\hat{y_{i}}-\bar{ŷ}\right)}{\sqrt{\sum_{i=1}^{T}{\left(y_{i}-ȳ\right)}^{2}}\sqrt{\sum_{i=1}^{T}{\left(\hat{y_{i}}-\bar{ŷ}\right)}^{2}}}, \end{array}$$

where ȳ and $\bar{ŷ}$ denote the sample mean of the chronological age and the predicted brain age, respectively. A non-parametric permutation test was applied to assess the statistical significance of above-chance predictive performance for the overall model as suggested in Hilger et al. (2020). In details, we permuted 1, 000 times the age outcomes of the subjects and assessed both performance values (MAE and R) within each permutation round. Finally, a *p*-value for each performance metric was assigned by dividing the number of times for which model performance based on the true age was lower than the performance for the permuted age outcomes by the number of permutations, i.e., 1,000.

### 3.2. Explainable Algorithms

In this work, we adopted the most popular local explanation algorithms: SHAP (Lundberg and Lee, 2017) and LIME (Ribeiro et al., 2016), to explain the decisions of the DNN models on each test sample. These methods are local model-agnostic as they explain predictions at individual level regardless the selected models. Basically, the two methods learn an interpretable linear model around each test instance and estimate feature importance at local level. For a dataset *D* = [(***x***_1_, *y*_1_), (***x***_2_, *y*_2_), ..., (***x***_*T*_, *y*_*T*_)], where $x_{i}\in {\mathbb{R}}^{P}$ is the feature vector for the sample *i* and *y*_*i*_ the corresponding age, the generic pre-trained model *f* returns a prediction *f*(***x***_*i*_) based on a single input sample ***x***_*i*_. SHAP and LIME aim at finding a linear model *g* to explain *f* by using a simplified inputs *x*′ that map the original inputs through a mapping function $x=h_{x}\left(x^{\prime }\right)$ trying to ensure $g\left(z^{\prime }\right)\approx f\left(h_{x}\left(z^{\prime }\right)\right)$ whenever *z*′ ≈ *x*′. Both methods minimize the following objective function:

(3) $$\begin{array}{l} \xi =\underset{g\in G}{arg min}L\left(f,g,\pi_{x^{\prime }}\right)+\Omega \left(g\right), \end{array}$$

where *G* is the class of linear models, $\pi_{x^{\prime }}$ represents a proximity metric between *x* and *x*′, Ω(*g*) denotes the complexity of the explanation *g* and the loss function L is defined as:

(4) $$\begin{array}{l} L\left(f,g,\pi_{x^{\prime }}\right)=\underset{x^{\prime }\in X^{\prime }}{\sum }{\left[f\left(x^{\prime }\right)-g\left(x^{\prime }\right)\right]}^{2}\pi_{x^{\prime }}, \end{array}$$

where *X*′ is the set of inputs within the neighborhood of *x*′. Both methods try to generate an explanation for *x* that approximates the behavior of the model accurately within the neighborhood of *x*, while achieving lower complexity (Slack et al., 2020). In other words, the methods explain the prediction of the instance *x* by computing the contribution of each feature to the prediction, so the absolute value of each SHAP and LIME value expresses how much each feature contributes to the final prediction (Wang et al., 2020). In LIME, Ω(*g*) and $\pi_{x^{\prime }}$ are defined heuristically, while in SHAP they are determined by satisfying some equations from the cooperative game theory. More details about the principles underlying these methods and the mathematical definitions can be found in the seminal works of Lundberg and Lee (2017) and Ribeiro et al. (2016). In our analysis, we applied the python implementation of the SHAP^4^ and LIME^5^ methods.

### 3.3. Reliability of Explainable Scores

Both SHAP and LIME are *post-hoc* local XAI methods as they exploit a pre-trained ML model to compute approximations of the model's inner decision logic by producing understandable representations in the form of feature importance scores for each independent test sample that represent the contribution of each feature to the final prediction of the ML model (Moradi and Samwald, 2021). These methods greatly differ from feature selection methods, which use the entire train set to determine the impact of each feature on a performance metric: the output of a feature selection scheme usually results in a single feature importance vector, whereas local XAI methods output a feature importance vector for each test sample. Therefore, a reliability analysis of XAI scores was performed to quantify the variation of the score values by slightly varying the composition of the training set. Moreover, since cognitive phenotypic variables and confounding factors related to the acquisition sites can affect the morphological feature values (Frangou et al., 2004; Shaw et al., 2006; Fortin et al., 2018), we investigated whether these factors could also influence the values and the reliability of the XAI scores.

**Figure 2:**
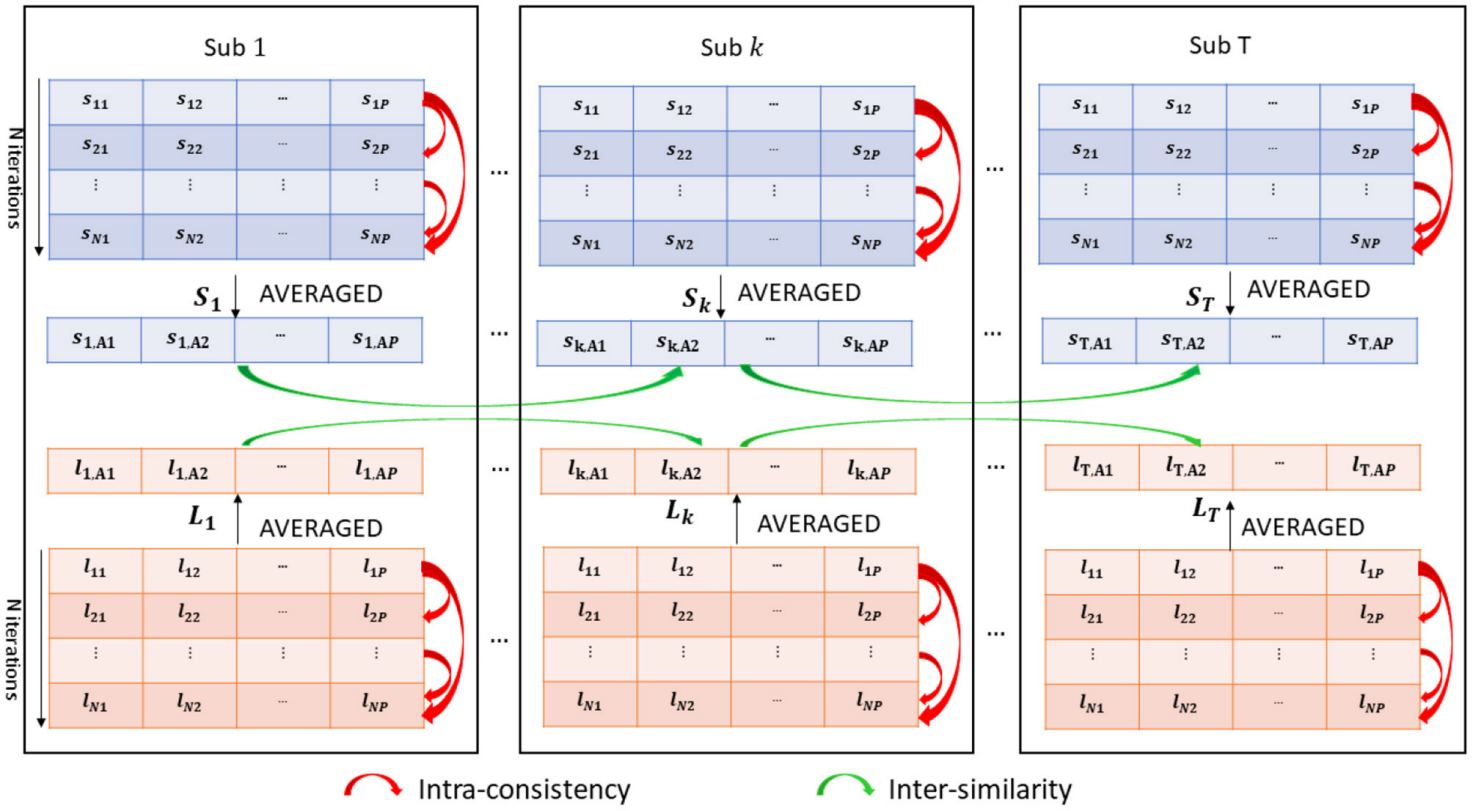
Analysis of intra-consistency and inter-similarity of both SHAP (blue) and LIME (orange) scores.

An overview of the methodology is shown in Figure 2. We collected *N* = 100 realizations of both SHAP and LIME vectors forming the two matrices *S* and *L* for each subject. We also averaged the *N* = 100 realizations of both values in order to obtain a single representative SHAP vector (*S*_*t*_ = [*s*_*t, A*1_, ..., *s*_*t,AP*_]) and LIME vector (*L*_*t*_ = [*l*_*t, A*1_, ..., *l*_*t,AP*_]) for each subject *t*, where:

(5) $$\begin{array}{l} s_{t,Ap}=\frac{1}{N}\overset{N}{\underset{n=1}{\sum }}s_{np} \end{array}$$

is the *p*^*th*^ averaged SHAP value for the feature *p*.

In order to investigated the reliability of both SHAP and LIME values, we computed:

- The intra-consistency coefficient of the scores, i.e., the correlation between each couple of score vectors ***s***_*k*_ = [*s*_*k*1_, *s*_*k*2_, ..., *s*_*kP*_] and ***s***_*z*_ = [*s*_*z*1_, *s*_*z*2_, ..., *s*_*zP*_], with *k, z* = 1, ..., *N* within each subject:
  (6) $$\begin{array}{l} IC_{kz}=\frac{\sum_{p=1}^{P}\left(s_{kp}-\bar{s_{k}}\right)\left(s_{zp}-\bar{s_{z}}\right)}{\sqrt{\sum_{p=1}^{P}{\left(s_{kp}-\bar{s_{k}}\right)}^{2}}\sqrt{\sum_{p=1}^{P}{\left(s_{zp}-\bar{s_{z}}\right)}^{2}}}, \end{array}$$
  where $\bar{s_{k}}$ and $\bar{s_{z}}$ denote the sample means of the two vectors and k and z denote the indices of different model training iterations. We also computed *IC*_*kz*_ for each couple of LIME vectors obtaining a distribution of $N\frac{\left(N-1\right)}{2}$ intra-consistency values for each XAI method. The intra-consistency coefficient varies between 0 (zero) and 1 (one), hence we compared the IC distributions by grouping the subjects according to their site. In addition, the correlation between the mean IC value of each subject and the variables age, FIQ and SNR was computed to verify if a possible association exists between the IC values of the subjects and each of the phenotypic information and imaging-related quality metric;
- The inter-subject similarity, i.e., the correlation between the SHAP (LIME) score vectors ***S***_*t*_ and ***S***_*u*_ (***L***_*t*_ and ***L***_*u*_) for each couple of subjects *u* and *t*, with *t, u* = 1, ..., *T*:
  (7) $$\begin{array}{l} IS_{ut}=\frac{\sum_{p=1}^{P}\left(s_{u,Ap}-\bar{S_{u}}\right)\left(s_{t,Ap}-\bar{S_{t}}\right)}{\sqrt{\sum_{p=1}^{P}{\left(s_{u,Ap}-\bar{S_{u}}\right)}^{2}}\sqrt{\sum_{p=1}^{P}{\left(s_{t,Ap}-\bar{S_{t}}\right)}^{2}}}, \end{array}$$
  where $\bar{S_{u}}$ and $\bar{S_{t}}$ denote the sample means of the two vectors. We then constructed an inter-similarity matrix *IS*, where entry (*u, t*) = *IS*_*ut*_ indicates the similarity value between the scores of subjects *u* and *t* for each of the two XAI methods obtaining matrices *IS*_*SHAP*_ and *IS*_*LIME*_. We applied the k-medoid algorithm on each *IS* matrix to find the best partition into clusters (more details on the algorithm are reported in Supplementary section 3 of Supplementary Material). The identified clusters of subjects were analyzed by using different criteria:
  1. The site membership to investigate a possible relationship between the XAI scores and the site of the subjects;
  2. The age, FIQ, and SNR distributions for comparing the phenotipic and imaging-related values across clusters. To analyze the differences between the identified clusters for each of the three variables, Kruskal–Wallis tests were conducted (α = 0.05 with Bonferroni corrections), followed by *post-hoc* Tukey–Kramer tests in case of significant group effects.

### 3.4. Comparison Between SHAP and LIME

We performed a direct comparison between the SHAP and LIME scores of each subject *t*, by computing the correlation between the SHAP and LIME vectors ***S***_*t*_ and ***L***_*T*_, with *t* = 1, ..., *T*:

(8) $$\begin{array}{l} R_{SL,t}=\frac{\sum_{p=1}^{P}\left(s_{t,Ap}-\bar{S_{t}}\right)\left(l_{t,Ap}-\bar{L_{t}}\right)}{\sqrt{\sum_{p=1}^{P}{\left(s_{t,Ap}-\bar{S_{t}}\right)}^{2}}\sqrt{\sum_{p=1}^{P}{\left(l_{t,Ap}-\bar{L_{t}}\right)}^{2}}}, \end{array}$$

where $\bar{S_{t}}$ and $\bar{L_{t}}$ denote the sample means of the two vectors.

In order to identify the set of morphological descriptors whose importance is most likely to vary with age, a correlation analysis was conducted between each of the SHAP and LIME averaged values and the age of the subjects. We considered α = 0.05 with Bonferroni corrections.

We also compared the set of most significant features between the two methods to verify a possible overlap between the two sets by means of the Jaccard coefficient:

(9) $$\begin{array}{l} J\left(A,B\right)=\frac{\left|A\cap B\right|}{\left|A\cup B\right|}, \end{array}$$

where *A* and *B* are two sets of significant features resulting from SHAP and LIME, respectively. The overlapping analysis was conducted by varying the threshold level between the 75*th* and 99*th* upper percentile and 1*st* and 25*th* lower percentile of the distributions of the correlation values with step Δ = 2. A non-parametric permutation test was performed by randomly permuting 1,000 times the correlation scores and assessing the percentage of overlap between the two sets to determine the statistical significance of the actual overlap for each threshold.

## 4. Results

### 4.1. Performance of DNN Models

**Figure 3:**
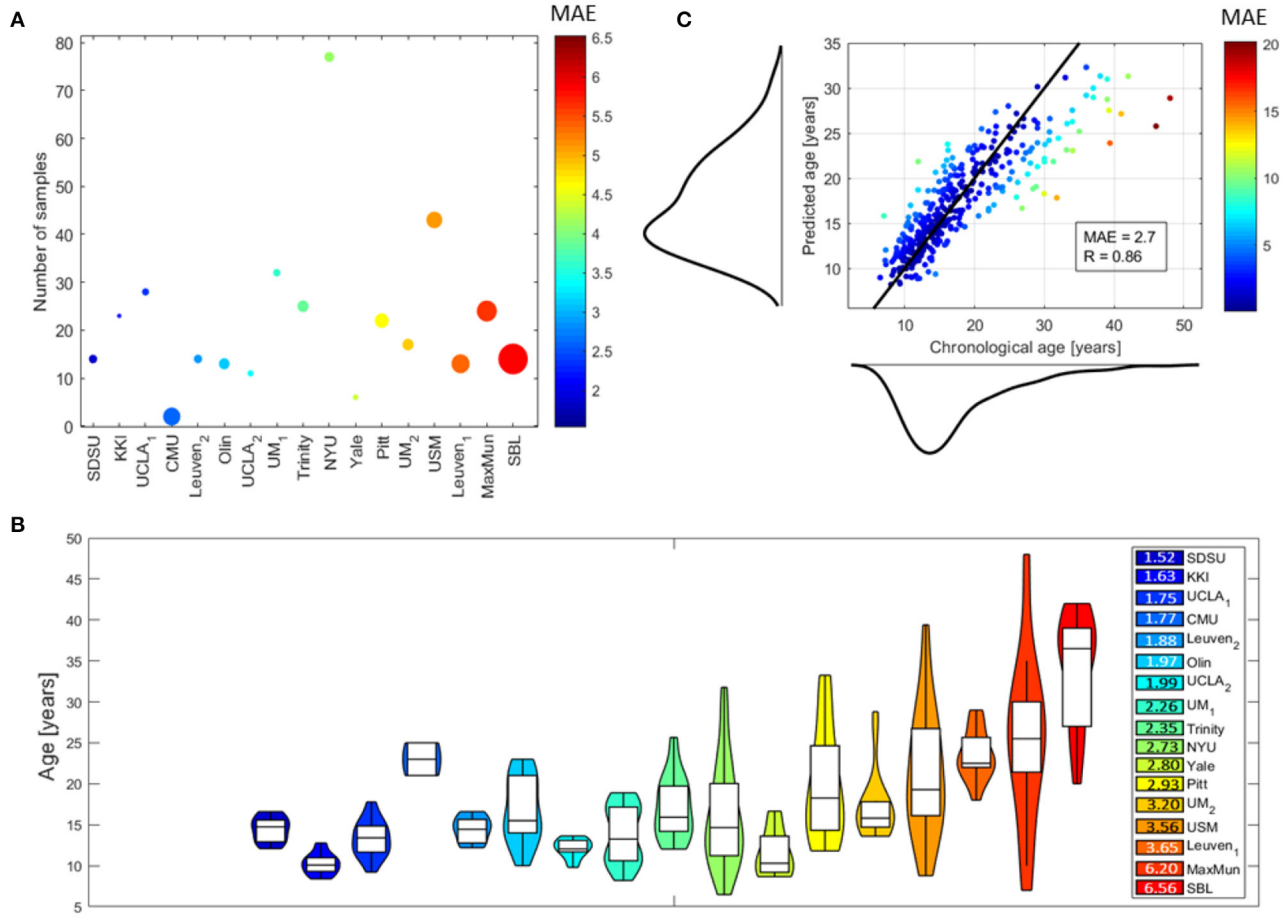
Performance of DNN models: **(A)** bubble plot where each bubble represents a site, the color codes the average MAE values of the subjects within each site and the radius is proportional to the average age of the subjects within each site, the y position indicates the number of samples within each site; **(B)** violin plots of the age distributions of the subjects within each site (the colors indicate the average MAE values for the subjects within each site); **(C)** scatter plot showing the chronological age of the subjects vs. their predicted age with the marginal distributions (the black solid line indicates the ideal model and the color of each point codes the average MAE for the corresponding subject). The performance for the whole dataset are also reported by means of the MAE and R-values.

Figure 3 shows the performance of the DNN models for the different sites and for each subject. In particular, Figure 3**A** shows a bubble plot reporting information on the average MAE for each site coded by colors, the number of samples along the y axis and the average age of the subjects within each site coded by the radius of each bubble. Figure 3**B** shows the violin plots of the age distributions of the subjects within each site sorted by increasing MAE coded by the same color map of Figure 3**A**. It is clearly evident from both plots that the MAE values are related to age range within each site: the higher the age range, the higher the average MAE within a site. Notably, the sites MaxMun and SBL which include subjects with age in the last percentile of the age distribution of the whole data samples (age >30 years) resulted the sites with the worst performance highlighting a sample size effect on this age range. This finding is better explained by inspecting Figure 3**C** which shows the averaged MAE for each subject in a scatter plot reporting also the chronological age and the predicted age with their marginal distributions: the chronological age distribution is highly right-skewed and the performance of the DNN models dramatically worse in the most sparse age range, i.e., the right tail of the distribution. For the whole dataset, we found the overall performance *MAE* = 2.7 and the correlation between the chronological and predicted age of the subjects *R* = 0.86. Both metrics were found to be significantly different from the chance level, resulting *p* = 0 from the non-parametric permutation test (see Supplementary Figure 1 for more details).

### 4.2. Intra-consistency of Explainable Scores

**Figure 4:**
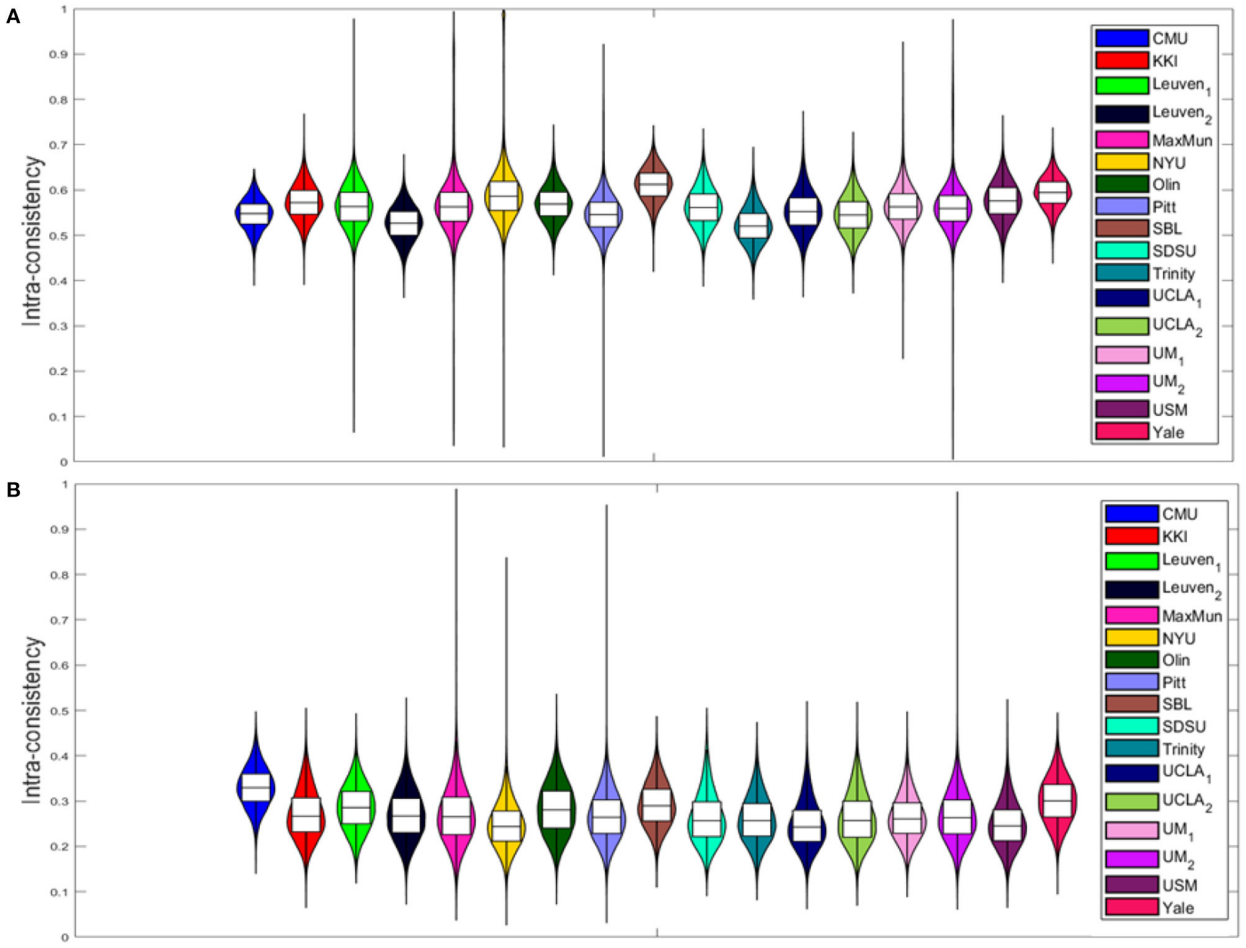
Violin plots of the intra-consistency distributions of **(A)** SHAP values and **(B)** LIME values for each site.

**Figure 5:**
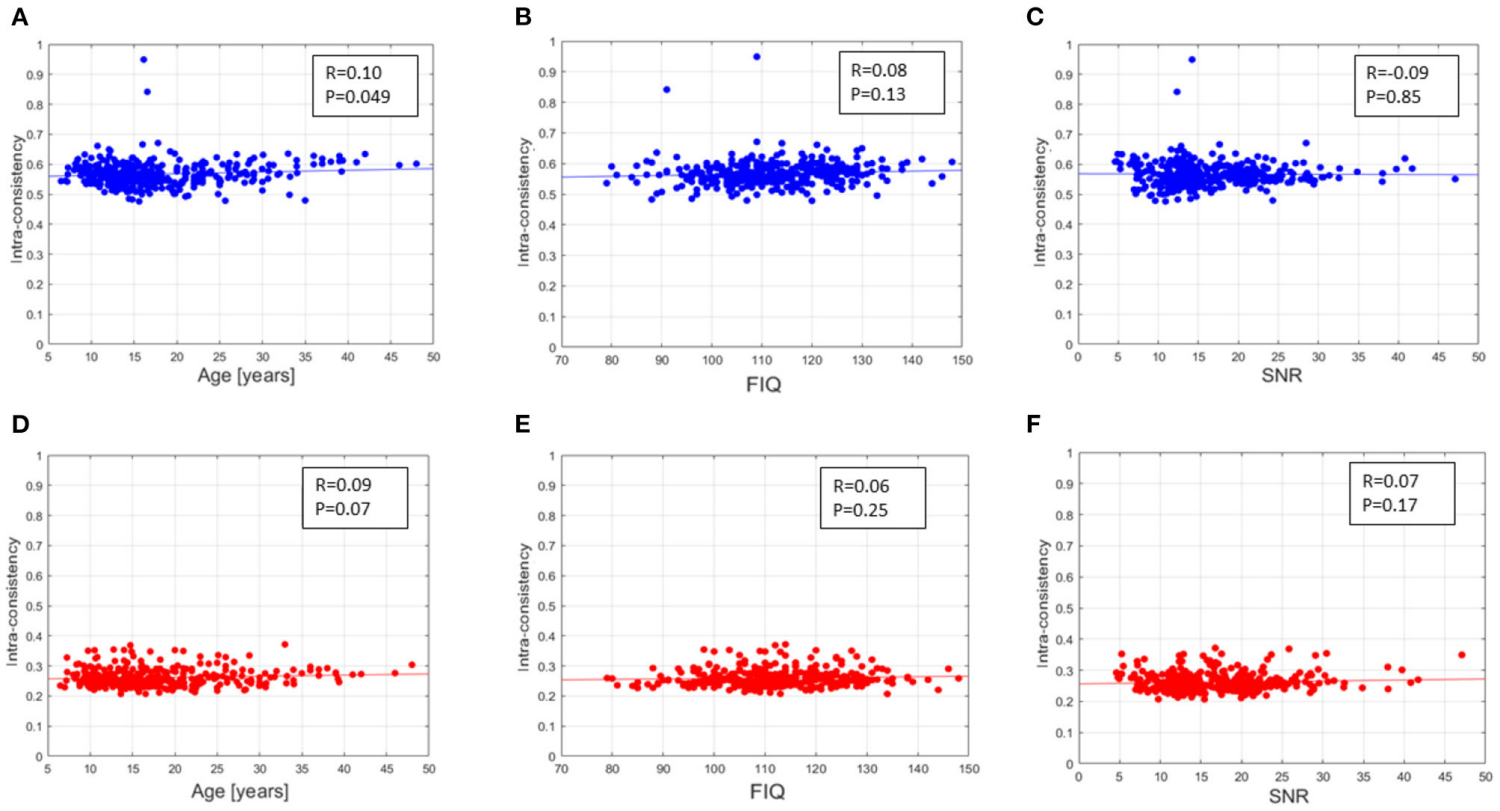
Correlation found between the averaged intra-consistency of SHAP values and **(A)** age, **(B)** FIQ and **(C)** SNR of the MRI of each subject; correlation found between the averaged intra-consistency of LIME values and **(D)** age, **(E)** FIQ and **(F)** SNR of the MRI of each subject.

The intra-consistency coefficients of the XAI scores provide indices of consistency of the feature importance as the training set varies from round to round. Figure 4 shows the distributions of these indices for the different sites for the SHAP scores (Figure 4A) and for the LIME scores (Figure 4B). Apart from a slight difference between the different sites for both scores, the LIME scores show consistently lower intra-consistency values (lower than 0.4 for all the sites) than those exhibited by the SHAP scores (greater than 0.5 for all the sites). Please refer to Supplementary Figure 2 for more details. We also evaluated the correlation between the averaged intra-consistency values and each of the age, FIQ and SNR variables to investigate whether any link exists between the average intra-consistency coefficients of both XAI methods and any of the biological, phenotypic and image-related characteristics of the subjects. Figure 5 shows the correlation between the averaged intra-consistency of the subjects and their age, FIQ and SNR for the SHAP method (Figures 5A–C) and LIME method (Figures 5D–F). Except for a weak correlation between the averaged intra-consistency indices of the SHAP values and the age of the subjects (*R* = 0.10, *P* = 0.049, not significant after Bonferroni correction), no significant correlations were observed for the other variables for both XAI methods.

### 4.3. Inter-similarity of Explainable Scores

**Figure 6:**
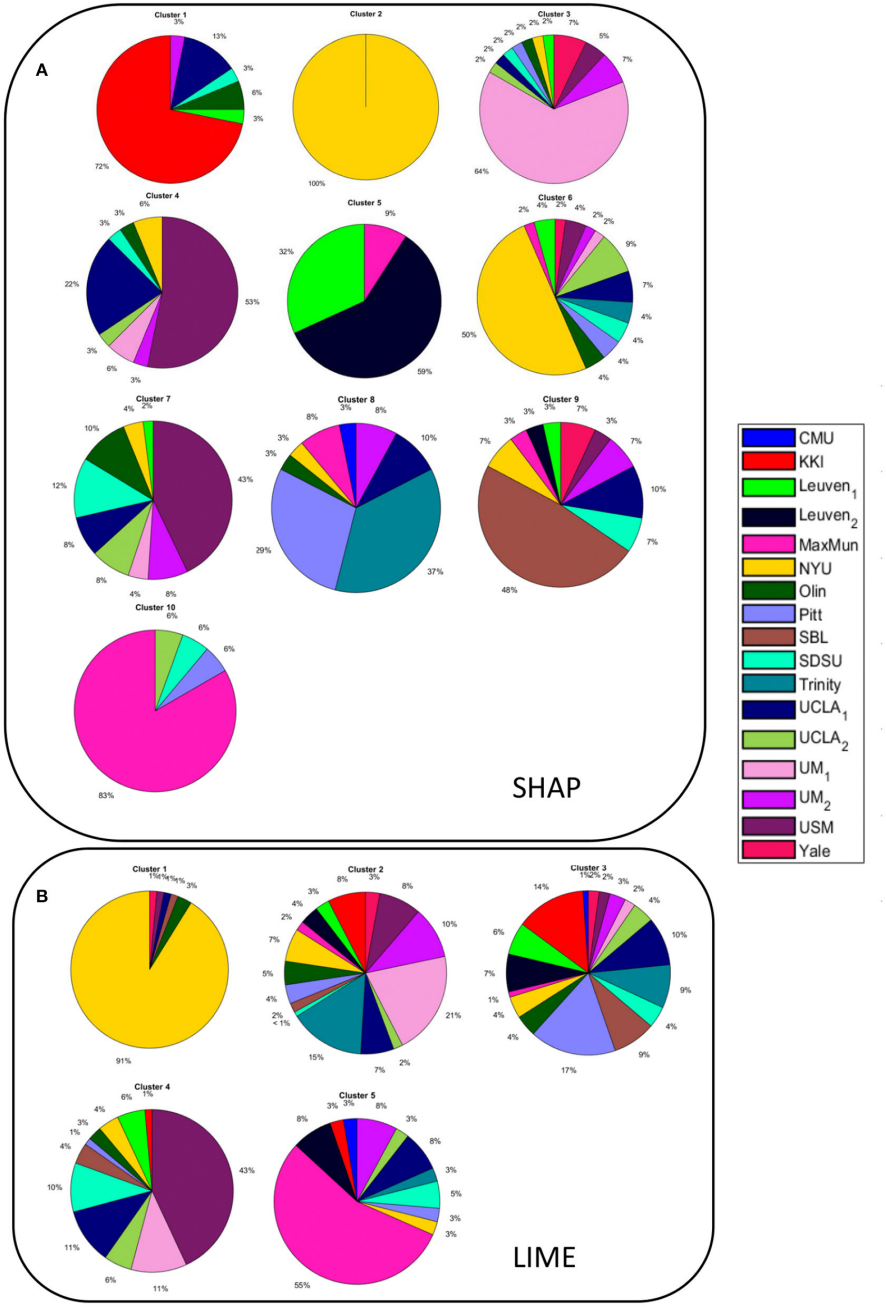
Pie charts showing the site membership of **(A)** the subjects belonging to each of the 10 clusters resulting from the partition of the inter-similarity network of the SHAP values; **(B)** the subjects belonging to each of the 5 clusters resulting from the partition of the inter-similarity network of the LIME values.

**Figure 7:**
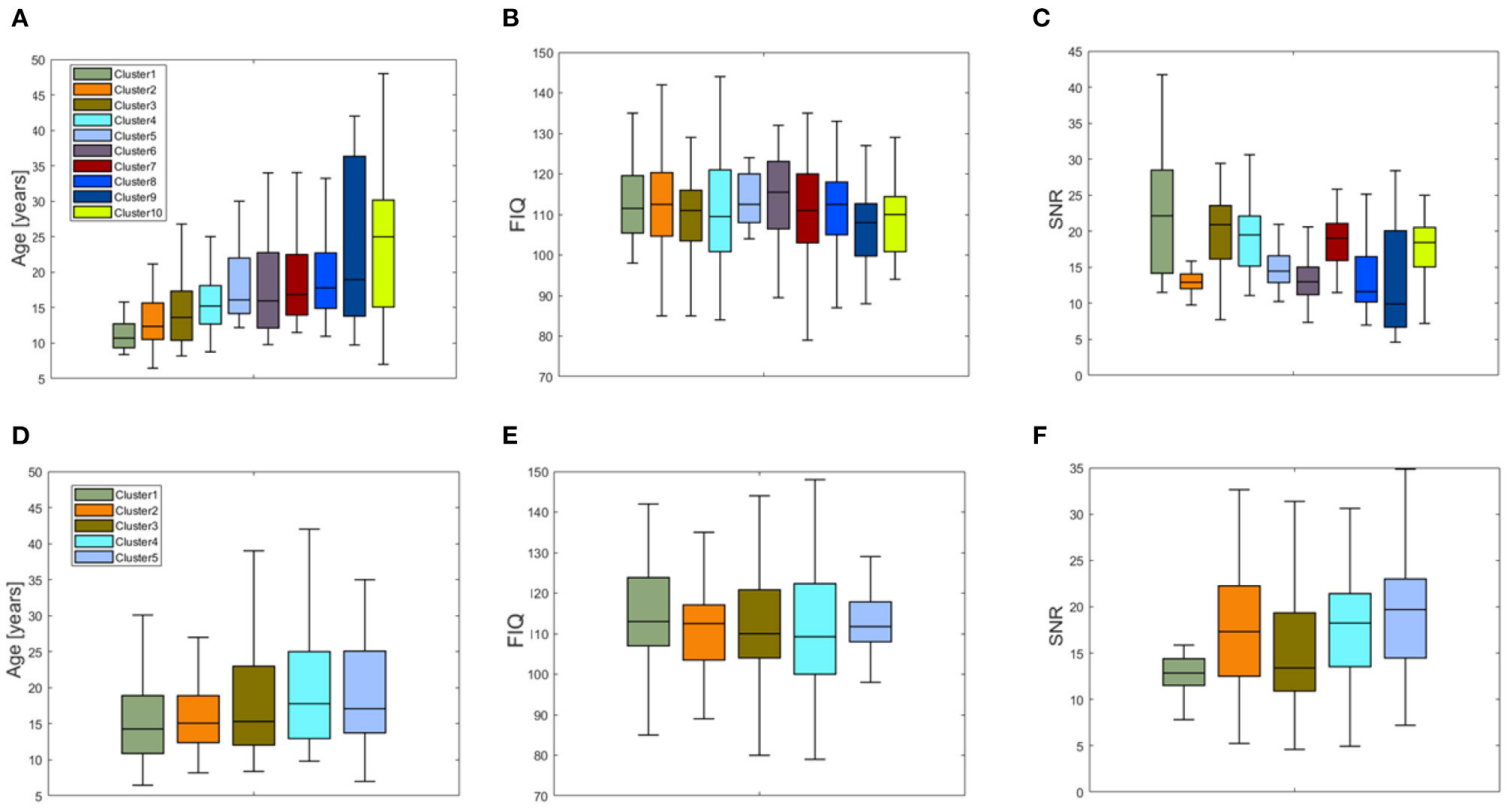
Boxplots of the distribution of **(A)** the age, **(B)** the FIQ values, and **(C)** SNR values for the 10 clusters resulting from the partition of the inter-similarity network of the SHAP values; boxplots of the distributions of **(D)** the age, **(E)** the FIQ values, and **(F)** SNR values for the five clusters resulting from the partition of the inter-similarity network of the LIME values.

We obtained two inter-similarity matrices (*IS*_*SHAP*_ and *IS*_*LIME*_) by computing the inter-similarity coefficient between each couple of average XAI score vectors of the subjects for each XAI method. The k-medoid method was used to assess the best partition of each *IS* matrix into clusters. We found *k* = 10 for matrix *IS*_*SHAP*_ and *k* = 5 for matrix *IS*_*LIME*_. More details on the clustering algorithm can be found in Supplementary section 3 of Supplementary Material. Figure 6 shows the pie charts reporting the site membership of the subjects within each of the 10 clusters for the SHAP values and five clusters for the LIME values. The different clusters are composed of individuals from different sites, apart from cluster 2 of the *IS*_*SHAP*_ matrix containing only individuals from the NYU site and cluster 1 of matrix *IS*_*LIME*_ composed mainly of subjects from the NYU site. We analyzed also the age, FIQ and SNR distributions of the subjects within each cluster of both inter-similarity networks. Figure 7 highlights that the age effect is greater in the *IS*_*SHAP*_ network as the age distributions of the different clusters differ more from each other (Kruskal–Wallis test: *p* < 10^−6^, Bonferroni corrected). The imaging quality was also detected as a strong effect in the *IS*_*SHAP*_ network as the SNR distributions of the clusters are significantly different (Kruskal–Wallis test: *p* < 10^−6^, Bonferroni corrected). More details on *post-hoc* tests are included in Supplementary section 4 of Supplementary Material.

### 4.4. Comparison Between SHAP and LIME

By directly comparing the SHAP and LIME vectors for each subject we found the average value *R*_*SL*_ = 0.52 ± 0.05, showing a weak correlation value between the two XAI scores.

**Figure 8:**
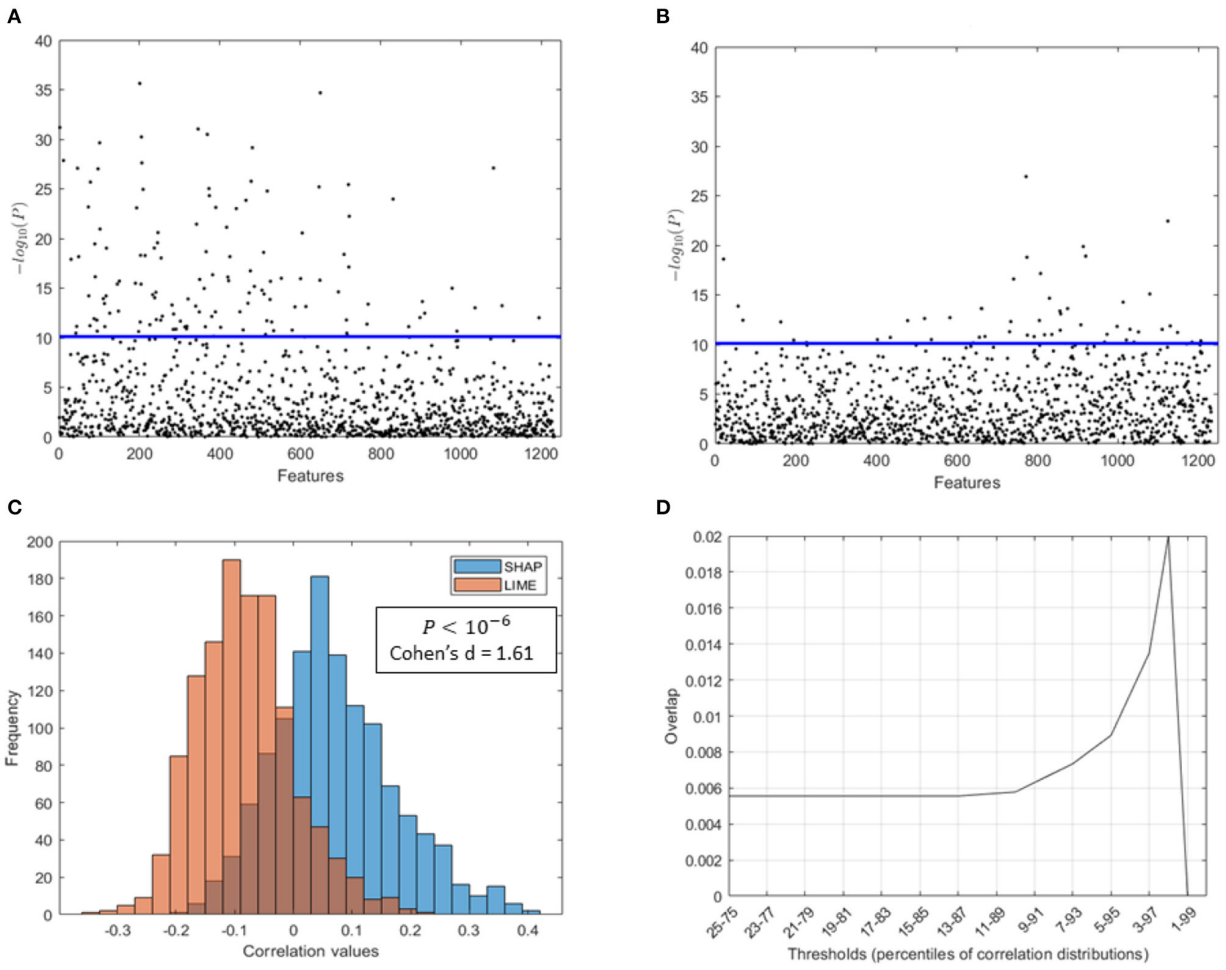
Comparison of SHAP and LIME scores. Manhattan plot representing the p values resulting from the correlation analysis between **(A)** the SHAP scores of the features and the age of the subjects, **(B)** the LIME scores of the features and the age of the subjects; **(C)** distributions of correlation values found between the SHAP/LIME scores of the features and the age of the subjects; **(D)** overlap between the most significant features for the two criteria (SHAP/LIME) selected by varying the percentile threshold of the correlation distributions.

**Figure 9:**
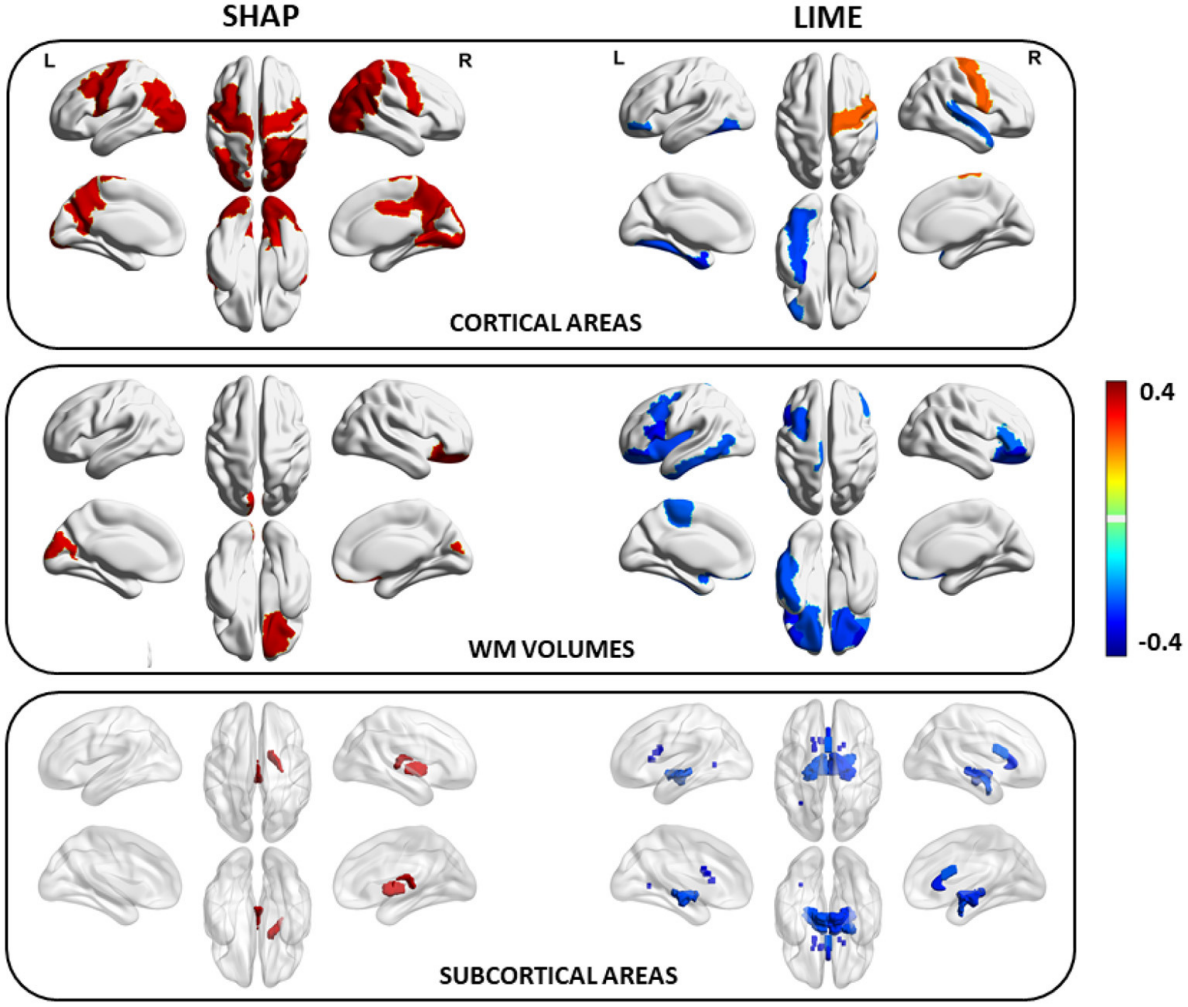
Brain regions resulting from the morphological features with the most significant correlation between the XAI scores and the chronological age for SHAP and LIME (the color of each ROI codes the average correlation value between the XAI scores of the features related to that ROI and the age of the subjects).

Figure 8 shows different results about the correlation analysis between the XAI scores and the age of the subjects:

- The distribution of coefficient values between the SHAP scores of the morphological features and the age of the subjects is significantly higher than the distribution of coefficient values between the LIME scores and the age (Wilcoxon test: *p* = 10^−6^; Cohen's *d* = 1.61) (see Figure 8C);
- A higher number of features statistically related to the age resulted from SHAP values than from the LIME values as presented in Figures 8A,B, which show the Manhattan plots representing the *p*-values resulting from the correlation analysis between the XAI scores of the features and the age of the subjects;
- The sets of age-related features for the two XAI methods exhibit a remarkably low overlap. Indeed, Figure 8D shows that for different threshold values of the two correlation distributions, the overlap coefficient between the two sets of features is below 0.02. This point is more obvious when comparing the two sets of morphological features with the most significant correlation between the XAI scores and the chronological age (at the threshold 2−98 percentiles of correlation distributions) for the two methods SHAP and LIME, listed in Tables 2, 3, respectively. A significant overlap was obtained for each threshold (*p* < 0.005). The brain regions related to the two sets of features are also represented in Figure 9. The two sets overlap only for one feature (*p* < 0.002), i.e., the curvature index of the right precentral ROI. Moreover, among the age-related features detected with the SHAP method, a prevalence of positively age-associated cortical features is reported, whereas a prevalence of negatively age-associated volumetric WM features is observed for the LIME method.

**Table 2:** The most significant age-related morphological features resulting from the SHAP scores grouped by category (R, Right; L, Left; curv, mean curvature; thick, thickness; vol, volume; the correlation coefficient for each feature is reported in brackets).

**Sub-cortical volume**	**Cortical features**	**WM volumes**	**Global features**
(+0.34) CSF normStdDev	(+0.35) L caudalmiddlefrontal CurvInd	(+0.33) wm L cuneus Vol	(+0.38) L mean thick
(+0.39) CSF normRange	(+0.33) L inferiorparietal ThickAvg	(+0.35) wm Rlateralorbitofrontal normMax	(+0.36) R mean thick
(+0.34) Right Pallidum normMin	(+0.34) L inferiorparietal CurvInd		
	(+0.35) L lateraloccipital ThickAvg		
	(+0.37) L lateraloccipital FoldInd		
	(+0.40) L precentral ThickAvg		
	(+0.37) L precentral FoldInd		
	(+0.35) L precentral CurvInd		
	(+0.34) L precuneus ThickAvg		
	(+0.38) R inferiorparietal ThickStd		
	(+0.37) R lateraloccipital ThickAvg		
	(+0.34) R lateraloccipital FoldInd		
	(+0.33) R lateraloccipital CurvInd		
	(+0.32) R lingual CurvInd		
	(+0.33) R posteriorcingulate ThickAvg		
	(+0.33) R precentral CurvInd		
	(+0.36) R precuneus ThickAvg		
	(+0.34) R superiorparietal CurvInd		

**Table 3:** The most significant age-related morphological features resulting from the LIME scores grouped by category (R, Right; L, Left; curv, curvature; thick, thickness; vol, volume; the correlation coefficient for each feature is reported in brackets).

**Sub-cortical volume**	**Cortical features**	**WM volumes**
(−0.23) L Cerebellum WM normMax	(−0.29) L entorhinal ThickAvg	(−0.25) L caudalmiddlefrontal normRange
(−0.25) L VentralDC normMax	(−0.25) L fusiform FoldInd	(−0.24) L inferiortemporal normMean
(−0.25) L VentralDC normRange	(−0.23) L parsorbitalis MeanCurv	(−0.24) L inferiortemporal normMin
(−0.24) R Amygdala normRange	(0.23) R precentral CurvInd	(−0.24) L lateralorbitofrontal normStdDev
(−0.27) R VentralDC Vol	(−0.23) R superiortemporal GrayVol	(−0.23) L paracentral normRange
(−0.35) non-WM hypointensities normMean		(−0.30) L parsopercularis normRange
(−0.30) non-WM hypointensities normMin		(−0.29) L parsorbitalis normRange
(−0.23) CC Mid Anterior normMax		(−0.25) L insula normStdDev
(−0.28) CC Anterior normMean		(−0.26) R lateralorbitofrontal normStdDev
		(−0.32) R parsorbitalis normRange
		(−0.22) R parstriangularis normRange

## 5. Discussion

In this work, we developed a novel XAI framework to perform brain age prediction with DNN and morphological features and compare the local explanations of the two XAI methods: SHAP and LIME. We adopted a cohort of healthy controls from ABIDE I dataset, whose heterogeneity is related to the different number of samples per site, the non-uniform age ranges per site and the various acquisition protocols. Hence, a leave-one-site cross validation strategy was chosen to investigate the site effect. Indeed, both the imaging and phenotipic heterogeneity of the data sample could affect the learning process and the final accuracy of the ML algorithms. The results the DNN models achieved compare favorably with the literature showing the overall performance *MAE* = 2.7 and *R* = 0.86 (Ball et al., 2019; Corps and Rekik, 2019,?; Zhao et al., 2019; Bellantuono et al., 2020). However, as shown in Figure 3, our models exhibit a systematic age under-estimation in the most extreme age-range of the distribution, reporting worse performance (*MAE* > 4) at sites with individuals with chronological age in that range. We found similar results in our previous work in which more simple ML models were applied on the same dataset (Lombardi et al., 2020b).

Afterwards, we applied the two XAI methods to each sample to derive the local explanations, i.e., a feature importance vector that express the contributions of the morphological features to the final prediction of the DNN models. We performed a hierarchical analysis to compare the reliability of the XAI scores both at subject-level and across subjects. Firstly, an intra-consistency score was defined to objectively assess the stability of the XAI methods for each subject with respect small variations of the training set. Indeed, the feature importance at local level should not vary significantly by slightly perturbing the composition of the training set in order to define a reliable personalized final ranking of the morphological features for the age prediction task (Kalousis et al., 2005; Lombardi et al., 2020a). We compared the intra-consistency values of both SHAP and LIME scores across the sites to verify a possible site effect on the feature importance. Figure 5, Supplementary Figure 2 and Supplementary Table 1 clearly highlight that some significant differences in intra-consistency values exist between some sites for both methods. Moreover, it is worth noting that the two XAI methods exhibit very different intra-consistency values as the SHAP intra-consistency scores are significantly higher than the LIME scores regardless the acquisition site. We computed the correlation coefficient between the averaged intra-consistency values of the subjects and each of the variables age, FIQ and SNR for both XAI methods to investigate the relationship between the feature importance and the phenotipic and imaging-related information. Figure 5 outlines that none of this variables is significantly related to the intra-consistency of both SHAP and LIME scores, hence the reliability of the XAI scores at local level does not depend on these characteristics of the subjects.

In order to compare the XAI scores across the subjects, we defined an inter-similarity score. We computed a single vector of SHAP and LIME scores for each subject by averaging the vectors resulting from the *N* under-sampling rounds of the training set. The purpose of this step was to obtain a single consistent feature importance vector for each method as by averaging the different realizations, the more stable scores are enhanced, while the more fluctuating scores are de-emphasized. We correlated the XAI vectors between each couple of subjects to assess the similarity of XAI scores among the subjects, i.e., the inter-similarity score. Finally, an inter-similarity matrix *IS* was constructed for each method and partitioned into clusters to detect groups of subjects with similar XAI scores. The analysis of the detected clusters show that except for a single cluster composed only by subjects from site NYU for the SHAP method and a cluster composed mainly of subjects from the NYU site for the LIME method, the other clusters include subjects from different sites (see Figure 6). This finding indicates that the feature importance values extracted by the two XAI methods also reflect the different characteristics of the NYU site with respect to all the other sites that have been discussed in several studies (Shehzad et al., 2015; Bhaumik et al., 2018). Similarly to the subject-based analysis, we compared the phenotypic and imaging quality-related variables between the different clusters for the two XAI methods. From Figure 7, it can be noted that only the clusters derived from the *IS*_*SHAP*_ matrix reflect a partition of the subjects into significantly different age ranges, whereas the clusters extracted from the *IS*_*LIME*_ matrix do not reveal a clear age-related partition of the subjects. This finding confirms the reliability of the SHAP scores with respect to the age prediction problem. Moreover, both partitions are related to the SNR of the subjects as the clusters also significantly differ for the imaging-related quality metric, showing that the site heterogeneity also affects the local XAI scores as well as the performance of the predictive models.

We performed a direct comparison between the SHAP and LIME vectors which highlighted a low correlation between the XAI scores for each subject. Moreover, a correlation analysis between each feature score vector and the age of the subjects was performed to yield a set of morphometric descriptors whose relevance for age prediction is most variable with age. This step of the framework provides global explanations of the DNN models since a set of age-related scores is extracted from the whole population under investigation. As shown in Figure 8C, the correlation distributions between the XAI scores and the age are markedly different from each other (*p* < 10^−6^, Cohen's *d* = 1.61). We reported the most age-related features for SHAP and LIME methods at the statistical threshold of the 97^*th*^ percentile of the correlation distributions in Tables 2, 3, respectively. The brain regions corresponding to the most age-related features for both XAI methods are shown in Figure 9. Average thickness, folding, and curvature index statistical attributes related to precentral gyrus and inferior and lateral occipital cortex were detected as the most age correlated for the SHAP method. Relevant morphological changes of these regions have been reported both in neurodevelopment and aging trajectories (Tamnes et al., 2010; McGinnis et al., 2011; Remer et al., 2017). In addition, changes in cortical curvature and folding of these regions have been extensively observed during brain maturation (Meng et al., 2014; Lefèvre et al., 2015). We also found CSF statistical descriptors as features significantly correlated with age in line with several works where cerebrospinal fluid biomarkers have been identified for normal aging process as well as for brain atrophy characterization (Preul et al., 2006; Baird et al., 2012; Vinke et al., 2018). In contrast, these regions do not appear among the most age-related LIME scores. In this set, features related to WM volumes of opercular and triangular part of inferior frontal gyrus and inferior temporal gyrus were detected as the most age-related descriptors. Notably, only the SHAP method showed a significant correlation between the importance of the cortical thickness of both hemispheres and age (*R* = 0.38 for left and *R* = 0.36 for right). This finding is highly consistent with several previous studies which, although reporting non-linear and widespread regional variations of both cortical and volume morphology with age, unequivocally agree on cortical thinning as a pattern of neurodevelopment (Zielinski et al., 2014; Fjell et al., 2015; Tamnes et al., 2017). In general, the age-related feature sets for the two methods strongly differ from each other as shown in Figure 8. Indeed, the overlapping analysis between the two sets highlights a low overlap, regardless the selected correlation threshold. In addition, a prevalence of negative age correlation values can be observed for the LIME scores. A significant negative correlation between the LIME values of a given morphological feature and the age of the subjects means that the LIME importance of that feature decreases as age increases. However, it is not possible to claim that the LIME scores better explain age in younger subjects than in older subjects as in our analysis we found that the LIME scores showed very low intra-consistency values regardless of the age of the subjects (as shown in Figure 5D).

## 6. Limitations and Future Directions

Although this work shows some important implications of SHAP and LIME XAI methods for the interpretations of brain age predictions with DNN models, it presents some limitations. We selected a cohort of typically neurodevelopment subjects from the ABIDE I dataset to explore the effect of heterogeneity of acquisition protocols and dataset composition on XAI scores. Our analysis revealed that the site effect also influences the XAI scores and therefore upstream harmonization techniques should be applied to the morphological features to reduce the batch effects (Fortin et al., 2018). Another important aspect concerning the selected cohort is its sample size and age range, indeed in our study morphological features are analyzed to predict the age of 378 subjects in the limited age range 6–48. Previous works have widely demonstrated that both the sample size and the age range could affect the performance of age prediction models (Amoroso et al., 2018; Peng et al., 2021). Moreover, currently the best state-of-the-art results have been achieved with datasets larger than 2,000 samples (Levakov et al., 2020; Peng et al., 2021). The reliability of the XAI values is closely related to the accuracy of the predictive models, so future developments will focus on training predictive models on larger cohorts with broader age range to extend the validity of our findings.

In this work we exploited a feature-based DNN age regression approach, therefore, we adopted SHAP and LIME to produce feature relevance morphological vectors as these two algorithms represent the two most established local model-agnostic XAI techniques. However, different XAI techniques have been developed to quantify the interpretability of the latent representations of CNNs: layer-wise Relevance Propagation (LRP) technique, saliency maps, and Gradient-weighted Class Activation Mapping (Grad-CAM) can be potentially used to produce coarse localization maps (Selvaraju et al., 2017; Eitel et al., 2019; Arrieta et al., 2020), highlighting the important regions in each MRI scan by exploiting the information at voxel level.

Finally, it is important to note that we performed a correlation analysis to identify the morphological descriptors whose importance most significantly varies with age. Hence, we compared the set of descriptors with the most significant correlation between the XAI scores and the age of the subjects to assess the overlap between the two XAI methods. However, a targeted and quantitative analysis is needed to compare the regions with significant impact on age prediction with the age-related regions reported in other studies. In future work, we will address a deeper comparison between the XAI scores of subjects grouped by age to existing meta-analysis.

## 7. Conclusion

In this work, we proposed a novel XAI framework to provide accurate explanations of both performance of DL algorithms and feature importance at subject level for age prediction with brain morphology. We extensively evaluated the reliability of the two XAI methods for the age prediction task both at subject level by assessing the intra-consistency of the XAI scores and across subjects by analyzing the inter-similarity of the scores. Our results reveal that the SHAP values showed significantly higher intra-consistency values than the LIME scores. This finding highlights that the SHAP scores are less influenced by small variations of the training set showing greater consistency of their values by varying the composition of the training set. Another interesting result concerns the analysis of the inter-similarity of the XAI scores between the subjects, which showed that the SHAP values more consistently reflect a partition of the subjects into different age ranges, proving therefore, a higher reliability of the SHAP scores for the age prediction task. The correlation analysis between the feature importance values and the age of the subjects showed that the two XAI methods detect totally different age-related features. In particular, the SHAP values exhibited a greater number of features statistically associated with age with higher absolute correlation values than those shown by the LIME method. Our findings indicate that the SHAP method could provide more reliable explanations for the morphological aging mechanisms that could be also exploited to identify personalized age-related imaging biomarkers.

## Data Availability Statement

Publicly available datasets were analyzed in this study. This data can be found here: http://fcon_1000.projects.nitrc.org/indi/abide.

## Author Contributions

AL and ST conceived the analysis. AL performed the data curation and performed the analysis. AL, DD, ST, and RB defined the methodology. AL and DD implemented the software pipelines and wrote the original draft. RB and ST supervised the analysis. AL, DD, ST, RB, NA, AM, and JT analyzed and interpreted the results and edited the final version of the manuscript. All authors have approved the final version of the manuscript.

## Conflict of Interest

The authors declare that the research was conducted in the absence of any commercial or financial relationships that could be construed as a potential conflict of interest.

## Footnotes

^1^http://fcon_1000.projects.nitrc.org/indi/abide

^2^https://www.recas-bari.it/index.php/en/

^3^https://surfer.nmr.mgh.harvard.edu/fswiki/recon-all

^4^https://anaconda.org/conda-forge/shap

^5^https://anaconda.org/conda-forge/lime
