# Supplementary material for: Explainable Deep Learning for Personalized Age Prediction With Brain Morphology
Source: Front Neurosci. 2021 May 28;15:674055. doi: 10.3389/fnins.2021.674055 (PMC8192966; doi:10.3389/fnins.2021.674055)
Supplement: Supplementary file 1 [file Data_Sheet_1.PDF]

Supplementary Material

1 STATISTICAL SIGNIFICANCE OF DNN PERFORMANCE

Figure S1 shows the histograms of the predictive performance (MAE and R) based on permuted data ($N = 1.000$ permutations; blue) in relation to the predictive performance based on the true non-permuted data (red vertical line). Both metrics resulted significantly different from chance level with $p = 0$.

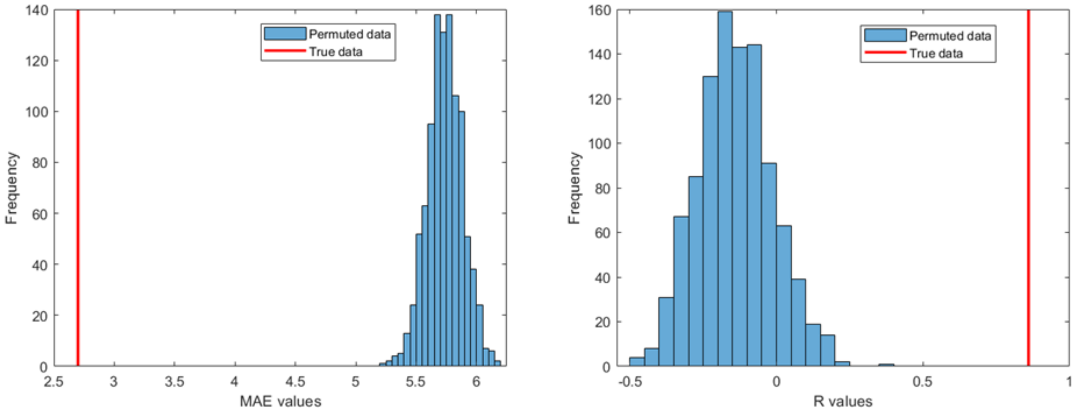

Figure S1. Histograms of the performance of the DNN models resulting from $N = 1.000$ permutations of the age outcomes compared to the actual overall performance (reported with red vertical lines).

2 COMPARISONS OF INTRA-CONSISTENCY COEFFICIENTS

Figure S2 shows the comparisons between the distributions of the IC values for the two XAI methods SHAP and LIME for each site. We compared the two distributions for each site by using the Wilcoxon rank-sum test. Table S1 reports p-values and Cohen's d coefficients resulting from the statistical tests.

3 IDENTIFICATION OF CLUSTERS

We applied the stability-based k-medoid criterion proposed by Hennig (2007) to find the best partition into clusters of the two inter-similarity matrices IS_{SHAP} and IS_{LIME}. This criterion assesses the clusterwise stability of a dataset by resampling it several times with different methods such as bootstrap or subsetting and by identifying the most stable clusters across the iterations. More in details in our analysis:

- the dataset is bootstrapped and a new dataset is created;
- the k-medoid algorithm is applied to find clusters; this step is performed for different number of clusters k and the Silhouette coefficients are computed;
- the number of clusters k is selected as the corresponding to the highest Silhouette coefficient (Rousseeuw (1987));
- for every given cluster in the original clustering the most similar cluster is searched in the new partition and the Jaccard similarity value is recorded;

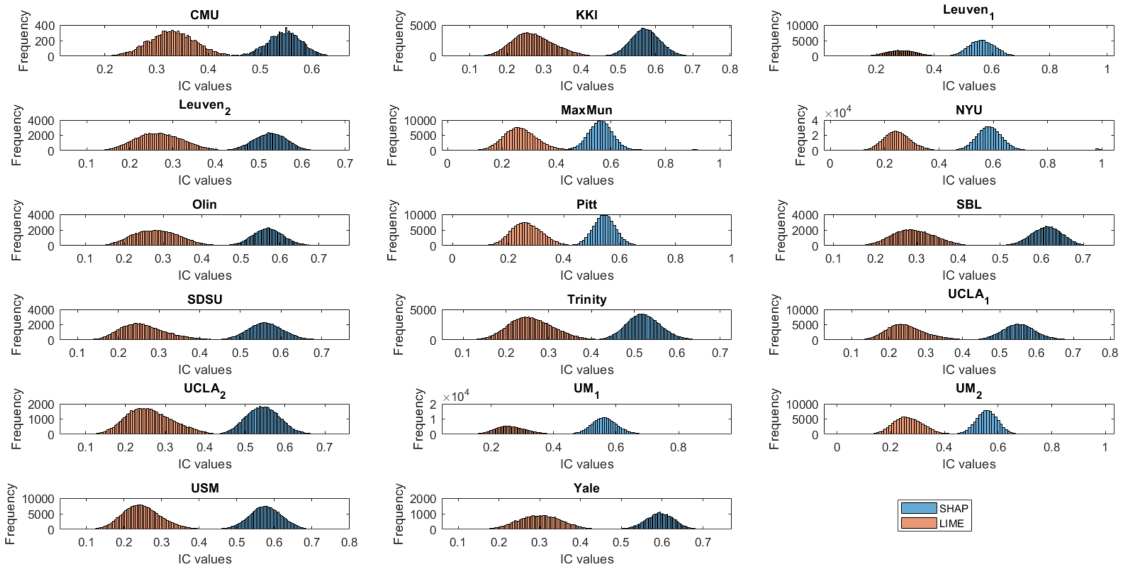

Figure S2. Histograms of the IC values for the SHAP and LIME methods for each site.

Site	P value	Cohen's d
CMU	10^{-7}	5.47
KKI	10^{-7}	6.31
Leuven 1	10^{-7}	4.91
Leuven 2	10^{-7}	5.46
MaxMun	10^{-7}	3.92
NYU	10^{-7}	5.38
Olin	10^{-7}	5.84
Pitt	10^{-7}	5.42
SBL	10^{-7}	7.24
SDSU	10^{-7}	5.84
Trinity	10^{-7}	5.58
UCLA 1	10^{-7}	6.13
UCLA 2	10^{-7}	5.61
UM 1	10^{-7}	6.15
UM 2	10^{-7}	4.76
USM	10^{-7}	6.92
Yale	10^{-7}	6.67

Table S1. Comparison between the distributions of the IC coefficients of the SHAP and LIME methods for each site: p-values resulting from the Wilcoxon rank sum test and Cohen's d coefficients.

- the cluster stability of each cluster is assessed by the average similarity taken over the I resampled datasets.

This algorithm returns the most stable k clusters, where k is automatically computed by using the quality of the partitions by means of the Silhouette coefficient. We used the R implementation "clusterboot"[1] of the algorithm by setting $I = 1000$ and $k = 1, 2, ..., 20$.

4 STATISTICAL COMPARISONS OF CLUSTERS

In this Section we report the results of the statistical comparison of the values of phenotypic and imaging variables between the clusters resulting from both IS_{SHAP} and IS_{LIME} matrices. We used the Kruskal–Wallis tests ($\alpha = 0.05$ with Bonferroni corrections), followed by post hoc Tukey-Kramer tests in case of significant group effects. Figure S3 shows the matrices of p-values from the post hoc Tukey-Kramer tests for the age and SNR variables.

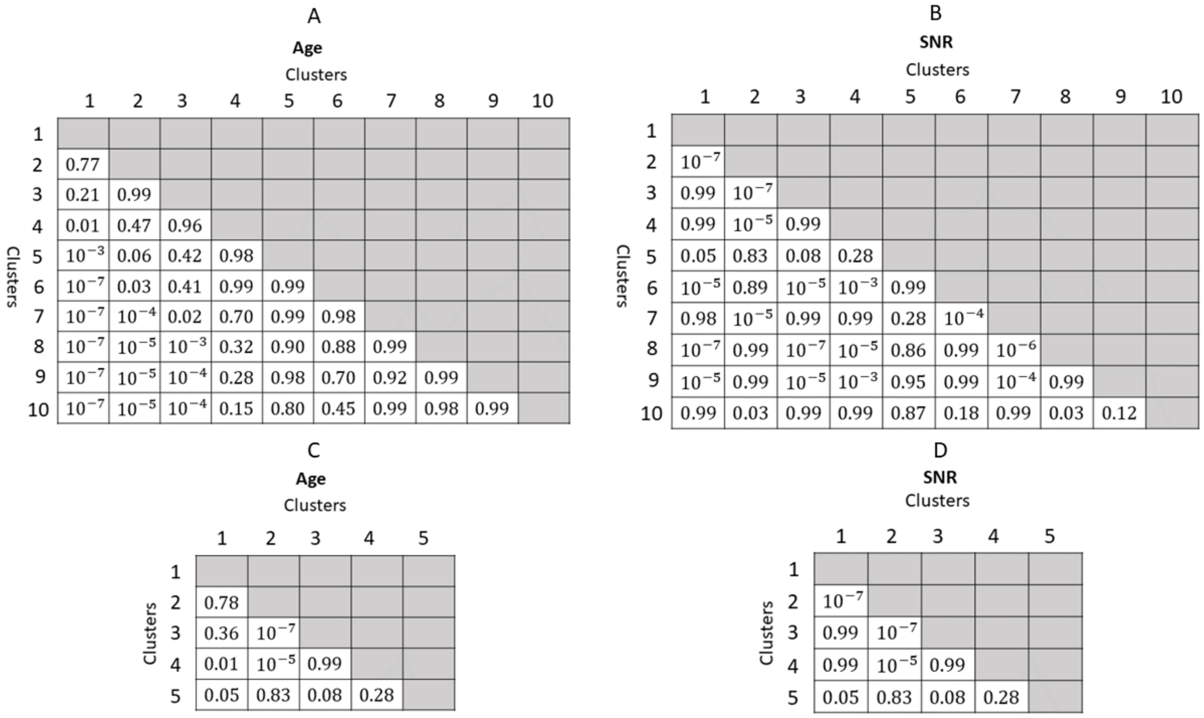

A
Age
Clusters

Clusters	1	2	3	4	5	6	7	8	9	10
1										
2	0.77									
3	0.21	0.99								
4	0.01	0.47	0.96							
5	10^{-3}	0.06	0.42	0.98						
6	10^{-7}	0.03	0.41	0.99	0.99					
7	10^{-7}	10^{-4}	0.02	0.70	0.99	0.98				
8	10^{-7}	10^{-5}	10^{-3}	0.32	0.90	0.88	0.99			
9	10^{-7}	10^{-5}	10^{-4}	0.28	0.98	0.70	0.92	0.99		
10	10^{-7}	10^{-5}	10^{-4}	0.15	0.80	0.45	0.99	0.98	0.99	

B
SNR
Clusters

Clusters	1	2	3	4	5	6	7	8	9	10
1										
2	10^{-7}									
3	0.99	10^{-7}								
4	0.99	10^{-5}	0.99							
5	0.05	0.83	0.08	0.28						
6	10^{-5}	0.89	10^{-5}	10^{-3}	0.99					
7	0.98	10^{-5}	0.99	0.99	0.28	10^{-4}				
8	10^{-7}	0.99	10^{-7}	10^{-5}	0.86	0.99	10^{-6}			
9	10^{-5}	0.99	10^{-5}	10^{-3}	0.95	0.99	10^{-4}	0.99		
10	0.99	0.03	0.99	0.99	0.87	0.18	0.99	0.03	0.12	

C
Age
Clusters

Clusters	1	2	3	4	5
1					
2	0.78				
3	0.36	10^{-7}			
4	0.01	10^{-5}	0.99		
5	0.05	0.83	0.08	0.28	

D
SNR
Clusters

Clusters	1	2	3	4	5
1					
2	10^{-7}				
3	0.99	10^{-7}			
4	0.99	10^{-5}	0.99		
5	0.05	0.83	0.08	0.28	

Figure S3. Matrices of p-values resulting from post hoc Tukey-Kramer test for **(A)** the age variable for the ten clusters of matrix IS_{SHAP}; **(B)** the SNR variable for the ten clusters of matrix IS_{SHAP}; **(C)** the age variable for the five clusters of matrix IS_{LIME}; **(C)** the SNR variable for the five clusters of matrix IS_{LIME}.

REFERENCES

Hennig, C. (2007). Cluster-wise assessment of cluster stability. *Computational Statistics & Data Analysis* 52, 258–271

Rousseeuw, P. J. (1987). Silhouettes: a graphical aid to the interpretation and validation of cluster analysis. *Journal of computational and applied mathematics* 20, 53–65

[1] `https://www.rdocumentation.org/packages/fpc/versions/2.2-9/topics/clusterboot`
